# Vitamin D Deficiency—Prognostic Marker or Mortality Risk Factor in End Stage Renal Disease Patients with Diabetes Mellitus Treated with Hemodialysis—A Prospective Multicenter Study

**DOI:** 10.1371/journal.pone.0126586

**Published:** 2015-05-12

**Authors:** Adalbert Schiller, Florica Gadalean, Oana Schiller, Romulus Timar, Flaviu Bob, Mircea Munteanu, Dana Stoian, Adelina Mihaescu, Bogdan Timar

**Affiliations:** 1 Department of Nephrology, ‘Victor Babes’ University of Medicine and Pharmacy, County Emergency Hospital, Timisoara, Romania; 2 B Braun Avitum Dialysis Center Timisoara, Timisoara, Romania; 3 Department of Diabetes and Metabolic Diseases, ‘Victor Babes’ University of Medicine and Pharmacy, County Emergency Hospital, Timisoara, Romania; 4 Department of Obstetrics and Gynecology, ‘Victor Babes’ University of Medicine and Pharmacy, County Emergency Hospital, Timisoara, Romania; 5 Department of Medical Informatics and Biostatistics, ‘Victor Babes’ University of Medicine and Pharmacy, County Emergency Hospital, Timisoara, Romania; University of São Paulo School of Medicine, BRAZIL

## Abstract

**Background:**

End stage renal disease (ESRD) patients on renal replacement therapy (RRT) with diabetes mellitus (DM) have a higher mortality rate and an increase prevalence of vitamin D deficiency compared to those without DM. It is still debated if vitamin D deficiency is a risk factor or a prognostic marker for mortality in these patients. This study investigated the prevalence of vitamin D deficiency and its impact on all-cause mortality in HD patients with DM.

**Methods:**

Our prospective non-interventional cohort study included 600 patients on hemodialysis therapy (HD) (median aged 56, interquartile range (19) years, 332 (55.3%) males) recruited from 7 HD centers, from all main geographical regions of Romania. The prevalence of DM was 15.3%. They were then followed regarding: dialysis duration, dialysis efficiency, renal anemia, CKD-MBD, inflammatory status and comorbidities: coronary artery disease (CAD), peripheral vascular disease (PVD) and stroke. The deficiency of 25-OH vitamin D was defined as a value lower than12 ng/mL.

**Results:**

Patients were followed for 3 years. The overall 3 year mortality was 25.5% (153 individuals), being higher in patients with DM as compared to those without DM (33.7% vs. 24.0%; P = 0.049). The time-related prognosis was also influenced by the presence of DM, at the survival analysis resulting in a HR of 1.52 [1.03 to 2.26] 95% CI, P = 0.037, for death in dialyzed patients with DM. In DM patients, 25-OH vitamin D deficiency was significantly higher (37.0% compared to 24.0%, P = 0.009). Furthermore, in patients with DM we observed a shorter dialysis duration (2 vs. 3 years, P<0.001) and a lower intact parathyroid hormone (iPTH) (258.0 pg/ml vs. 441.9 pg/ml, P = 0.002). Regarding the presence of comorbidities at the inclusion in the study, the presence of diabetes in dialyzed patients was associated with increased prevalence of CAD (87.0% vs. 58.1%, P<0.001), PVD (67.4% vs. 17.3%, P<0.001) and history of stroke (29.3% vs. 14.0%, P<0.001). In patients with DM the presence of 25-OH vitamin D deficiency increased the probability of death (50.0% vs. 24.1%; P = 0.011). In multiple Cox proportional hazards analysis, vitamin D deficiency remained an independent predictor for mortality in dialysis patients with DM (HR = 1.71, 95% CI 1.21 to 2.43, P = 0.003). In the same time, multiple Cox proportional hazards analysis showed that age (HR = 1.02 per one year increase, P = 0.004), CAD (HR = 1.55, P = 0.046) and PVD (HR = 1.50, P = 0.029) were independent predictors for mortality in dialysis patients with DM.

**Conclusions:**

ESRD patients with DM treated with HD have a higher overall mortality than non-DM patients. Vitamin D deficiency is significantly more prevalent in HD patients with DM. Low 25-OH vitamin D levels were associated with increased all-cause mortality in these patients. According to our data, in HD patients with DM, screening for vitamin D deficiency (and its correction) should be mandatory for an optimal risk reduction strategy.

## Introduction

The number of end stage renal disease (ESRD) patients on renal replacement therapy (RRT) is increasing all over the world, diabetes mellitus (DM) being the leading cause. In the last decade the prevalence of ESRD attributed to diabetic kidney disease (DKD) increased 2.5 fold [1, 2]. On the other hand, ESRD patients treated with hemodialysis (HD) have a 7 fold higher mortality rate as compared to the general population and in ESRD-DM population the mortality increases even more. According to 2013 USRDS data only 50% of the ESRD-DM patients on HD are surviving at 3 years and only 30% are alive at 5 years of therapy [1]. Increased prevalence of traditional cardiovascular disease (CVD) risk factors in ESRD patients and CVD mortality do not entirely explain the very high all-cause mortality rate of these patients. It is well established that in CKD patients, the Framingham risk equation, which estimated cardiovascular disease risk based on traditional risk factors (i.e. age, gender, diabetic status, smoking status, serum total cholesterol level, systolic blood pressure, and left ventricular hypertrophy by electrocardiography) is insufficient to predict all of the cardiovascular disease risk in CKD patients [3, 4,5].

Chronic kidney disease related mineral bone disorder (CKD-MBD), (i.e. anomalies of calcium, phosphate, intact parathyroid hormone (iPTH), vitamin D, vascular and heart valve calcifications) was lately also related to the high mortality rate. In the general population and in CKD patients vitamin D deficiency was associated with elevated cardiovascular (CV) morbidity, mortality and all-cause mortality [6,7].The prevalence of vitamin D anomalies is increased both in the general population and even more in CKD patients (25-OH vitamin D is considered the standardized biomarker for vitamin D status and typically, vitamin D deficiency is defined as circulating 25—OH vitamin D levels<25 nmol/L, respectively vitamin D insufficiency is defined as circulating 25—OH vitamin D levels between 25 to 50 nmol/L, (to convert to ng/ml, divide by 2.496) [6, 7, 8, 9]. In patients with CKD stage 5 on dialysis, the prevalence of vitamin D anomalies may go up to 90% [10] and is associated with increased arterial stiffness [11], increased prevalence of vascular calcifications [12], stroke [13], LVH [14] and increased risk of all cause and cardiovascular mortality [13].

It seems that the prevalence of vitamin D anomalies is even higher in DKD [15, 16]. The low vitamin D levels are associated with increased mortality in patients with mild or moderately reduced kidney function in both type 1 and 2 DM [17, 18]. Less data are available about ESRD-DM patients treated with HD and vitamin D deficiency [13]. The aim of the present study was to assess the prevalence of vitamin D deficiency and its relationship with risk of all-cause mortality in HD patients with DM.

## Materials and Methods

In November 2010, 600 ESRD patients (332 men and 268 women) treated with HD in 7 centers from Romania were enrolled in this prospective, observational study. At inclusion, patient’s data have been retrieved from their medical records: personal data (age, gender), medical history (etiology of CKD, coronary artery disease—CAD, peripheral vascular disease—PVD, stroke, DM), dialysis related data (duration of dialysis therapy, previous 6 months average eKt/V, duration and number of dialysis sessions/week, type of dialyzer, Qb), anthropometric data (height, weight, body mass index-BMI).The initial laboratory work-up included: hemoglobin, ferritin, transferrin saturation (TSAT), C-reactive protein (CRP), albumin, Ca, PO4, calcium-phosphate product (CaxPO4), HCO3-, iPTH, alkaline phosphatase (ALP), 25-OH vitamin D, alanine aminotransferase (ALAT), aspartate aminotransferase (ASAT), hepatitis B virus (HBV) and hepatitis C virus (HCV) infection. Patients were treated with high flux, high surface, polysulfone (Xevonta) filters (not reused) and ready-to-use dialysis fluid (B. Braun acidic bicarbonate hemodialysis concentrate). Renal anemia and CKD- MBD was treated according to the KDIGO guidelines. The patients were followed-up three years after their enrollment or death. No patient was lost to follow-up.

25-OH vitamin D was measured only once at initial evaluation by chemiluminescente immunoassay with an inter assay coefficient of variation of 5.5–9.2% from blood samples drawn in November, before the start of dialysis session. We couldn’t use the liquid chromatography method which is considered the gold standard techniques for circulating levels of vitamin D assessment. Actually, there is still no consensus about the cut-off values for 25-OH vitamin D deficiency. According to The Institute of Medicine (IOM) vitamin D deficiency was defined as 25-OH vitamin D levels below 12 ng/ml [19]. The IOM commitee suggests that people are at risk of deficiency related to bone health at serum 25-OH vitamin D levels of below 30 nmol/L (12 ng/mL) [19]. It seems that IOM cut-off for vitamin D deficiency is also appropriate for mortality prediction [20]]. On the other hand, Holick et al. have suggested that vitamin D deficiency should be defined as 25-OH vitamin D below 20 ng/ml [21]. Using higher than appropriate cut-off levels for serum 25-OH vitamin D would artificially increase the estimates of the prevalence of vitamin D deficiency [19]. Therefore, in the present study we considered for 25-OH vitamin D deficiency, the cut-off values proposed by, IOM respectively 25-OH vitamin D levels below 12 ng/ml [19]. At the baseline of the present study, all the patients were screened for diabetes. A dialysis patient was considered to have DM if diabetic nephropathy was the primary cause of ESRD, or if diabetes was present as co-morbidity. Those patients who developed DM over the follow-up period of 3 years, were not included in the DM group and continued to be part of the non-DM group.

### Statistical analysis

Data were collected and analyzed using the SPSS v.17 software suite (SPSS Inc. Chicago, IL, USA) and are presented as mean ± standard deviations for continuous variables with Gaussian distribution, median (interquartile range) for continuous variables without Gaussian distribution, or percentages for categorical variables. The lower and upper limits of the 95% confidence intervals (CI), used to estimate the prevalence, were calculated according to Wilson’s procedure for variables with Poisson distribution. Moreover, the 95% CI for odds ratio (OR) was calculated according to the mid-p method for binomial distributions. Survival was analyzed with Hazard Ratio (HR) method and presented using Kaplan-Meier diagrams. To assess the significance of the differences between groups, the Student *t*-test (means, Gaussian populations), Mann-Whitney-U test (medians, non-Gaussian populations), Chi-square (proportions) and log-rank test (differences between survival curves and hazard ratio) were used. Continuous variable distributions were tested for normality using Shapiro-Wilk test, and for equality of variances using Levene’s test. For evaluating the involvement of more confounding factors in dichotomous outcomes, multivariate logistic regression models were built, their goodness of fit being evaluated using Hosmer-Lemeshov method. For evaluating the involvement of more confounding factors in time-related risk, Cox proportional-hazards models were built. A p value of <0.05 was considered as the threshold for statistical significance.

The studied group baseline characteristics are presented in Table 1.

**Table 1:** Baseline characteristics of the studied group.

Studied parameter	Result
Men (%)	332 [55.3%)
Age (years)^a^	56 (19)
Time from first hemodialysis session (years)^a^	2.8 (5)
Weekly hemodialysis time (hours)^a^	12 (3)
eKt/V^b^	1.35 ± 0.46
BMI (kg/ m^2^) ^b^	25.4 ± 4.8 kg/m^2^
CAD (%)	375 (62.5%)
PVD (%)	150 (25.1%)
Stroke (%)	98 (16.3%)
Diabetes Mellitus (%)	92 (15.3%)

^a^ Distributions are not Gaussian. Data is presented as median and [interquartile range]

^b^ Data is presented as mean±SD

BMI—body mass index; CAD-Coronary artery disease; PVD—Peripheral vascular disease

The studied group was divided in three cohorts according to the presence of diabetes and respectively to the deficiency of 25-OH vitamin D as following: dialyzed patients without diabetes, with DM and normal 25-OH vitamin D and patients with DM and 25-OH vitamin D deficiency.

### Ethics statement

The study was approved by the BBraun Avitum Ltd Romania Ethical Committee (Board of Human Studies) and every patient provided written informed consent before enrolment.

## Results

The prevalence of DM in the studied cohort was 15.3% (92 patients). The patients who developed DM after enrollment in this study continued to be included in non-DM group. The reason for this decision was the fact that very short time from DM onset could not significantly alter mortality results, being well-established that duration of diabetes represents one factor that plays an important role in determining the risk of death in people with diabetes [22]. Vitamin D deficiency was identified in 37.0% of the DM patients ([25.6% to 51.6%] 95% CI), being significantly more prevalent as compared to non- diabetics 24.0% ([19.9% to 28.7%] 95%CI), (p = 0.009). DM patients had a shorter HD duration (2 vs. 3 years, p<0.001), lower iPTH (258.0 vs. 441.9 pg/ml, p = 0.002) and 25-OH vitamin D levels (median value: 15.0 vs. 21.0 ng/ml, p = 0.001). Marginally significant differences were observed for age (59 vs. 56 years, p = 0.078), albumin (3.7 vs. 3.9 g/dL, p = 0.052) and BMI (26.3 vs. 25.3 kg/m^2^, p = 0.079). The other studied parameters, did not significantly differ in the two groups. (Table 2).

**Table 2:** Comparison between DM and no DM groups.

Parameter	Without DM (n = 508)	With DM (n = 92)	p
Age (years) ^a^	56 [17]	59 [13]	0.078
Dialysis duration (years) ^a^	3 [5]	2 [2]	<0.001*
Weekly dialysis time (hours) ^a^	12 [1.5]	12 [1.6]	0.064
eKtV ^b^	1.35 ± 0.46	1.32 ± 0.46	0.58
BMI (kg/m^2^) ^b^	25.3 ± 4.9	26.3 ± 4.8	0.079
Hemoglobin (g/dL) ^b^	11.2 ± 1.6	11.3 ± 1.5	0.55
Ferritin (ng/mL) ^a^	549.5 [514.3]	498.0 [422.0]	0.165
TSAT (%)^a^	19.0 [29.7]	15.0 [33.3]	0.297
hsCRP (mg/dL)^a^	2.0 [4.0]	2.0 [6.0]	0.096
Albumin (g/dL) ^b^	3.9 ± 0.7	3.7 ± 0.7	0.052
Ca (mg/dL) ^b^	8.5 ± 1.1	8.4 ± 0.9	0.433
PO4 (mg/dL) ^b^	5.7 ± 1.7	5.6 ± 1.7	0.480
CaxPO4(mg^2^/dl^2^) ^b^	48.9 ± 15.5	47.4 ± 14.4	0.957
HCO3(mmol/L) ^b^	19.3 ± 4.4	20.2 ± 4.4	0.099
iPTH (pg/ml) ^a^	441.9 [663.0]	258.0 [476.3]	0.002*
25-OH vitamin D (ng/ml) ^a^	21.0 [21.0]	15.0 [16.5]	0.001*
ALP ^a^	96.5 [63.3]	99.5 [73.5]	0.799
Calcitriol supplementation (%) ^c^	120 (23.6%)	58 (26.1%)	<0.001*

*Differences are significant

^a^ Distributions are not Gaussian. Data is presented as median and [interquartile range]

^b^ Data is presented as mean±SD

^c^ Data is presented as number and (percentage of total). p was calculated using chi-square test.

BMI—body mass index; ALP—alkaline phosphatase, TSAT- transferrin saturation, hsCRP-high sensitive C-reactive protein

Patients with DM treated with HD presented a significantly higher prevalence of CAD (87.0% vs. 58.1%, p<0.001), of PVD (67.4% vs. 17.3%, p<0.001) and history of stroke (29.3% vs. 14.0%, p<0.001). The prevalence of hepatitis B and C virus infection did not significantly differ in the two groups. There were also no differences between the groups regarding anemia therapy (erythropoietin stimulating agents, and iron) and CKD-MBD treatment (paricalcitol, vitamin D3, use and type of phosphate binders).

According to our model, the presence of DM was associated with vitamin D deficiency, however it is known that several other factors, also associated with DM might be involved in generating the observed vitamin D deficiency, like the presence of obesity, higher age or the presence of other comorbidities, like CAD or PVD. To analyze if the DM is also an independent factor involved in generating the vitamin D deficiency, we created a multivariate logistic regression model having as outcome the vitamin D deficiency and as predictors the age, BMI, presence of DM, CAD and PVD. The results of the model are presented in Table 3.

**Table 3:** Multivariate logistic regression model.

Predictor	B	Exp (β)	p
Age (years) ^a^	0.023	1.023	0.004*
BMI (kg/m^2^) ^a^	-0.009	0.991	0.678
DM (dichotomous) ^b^	0.254	1.289	0.037*
CAD (dichotomous) ^b^	0.613	1.848	0.010*
PVD (dichotomous) ^b^	0.151	0.092	0.535

* Factors with independent impact in generating vitamin D deficiency, after adjusting for confounding factors

^a^ Predictors were added in the model as continuous, scale measured variables

^b^ Predictors were added in the model as dichotomous variables

BMI—body mass index; CAD—Coronary artery disease; DM—diabetes mellitus; PVD—Peripheral vascular disease

According to the results of our model the presence of DM along with age and the presence of CAD were significant factors which acted independently in generating vitamin D deficiency in our studied cohort, even after correcting for other possible confounding factors.

**Fig 1:**
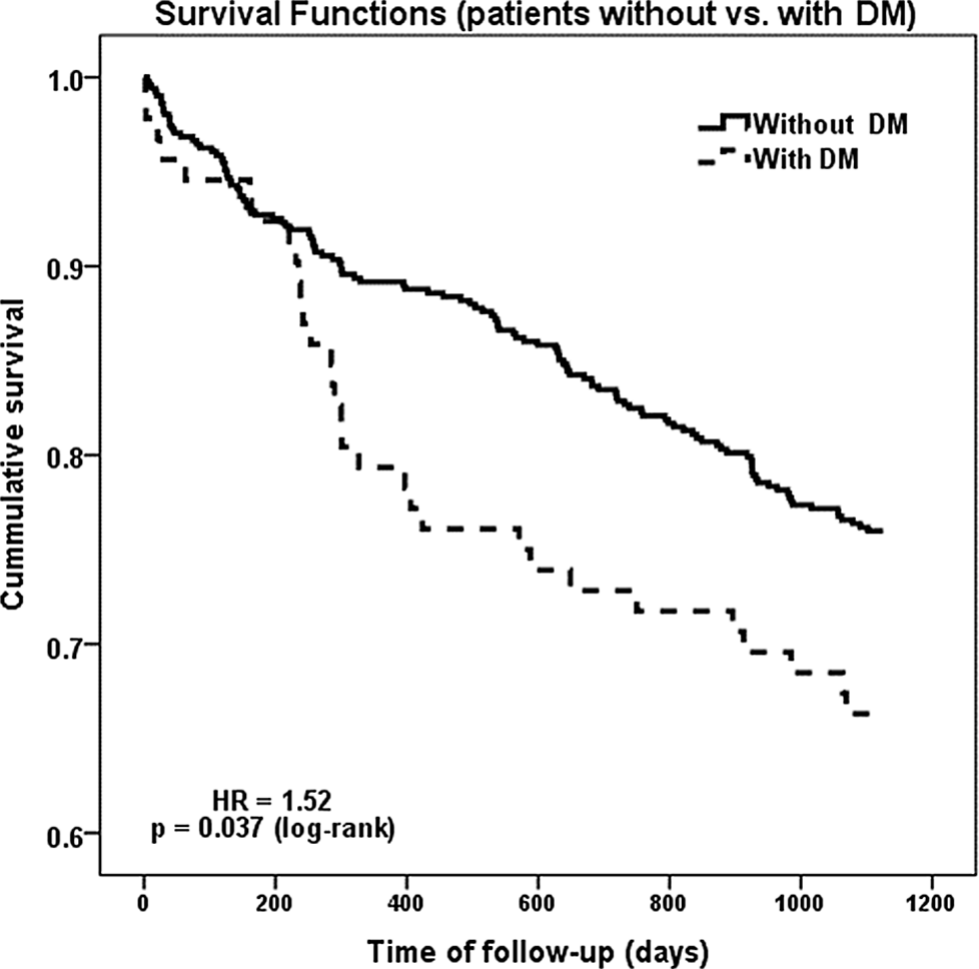
Survival analysis: dialyzed patients with DM vs. without DM.

In our cohort of patients the overall mortality in the three years of follow up was 25.5% (153 individuals), being higher in patients with DM (33.7% vs. 24.0%; p = 0.049). The time-related prognosis was also influenced by the presence of DM. The survival analysis revealed a Hazard Ratio (HR) of 1.52, [1.03 to 2.26]95%CI, p = 0.037 for death in dialyzed patients with DM compared to non-diabetics, (Fig 1).

The results of comparative evaluation of subgroup of DM patients without 25-OH vitamin D deficiency versus subgroup of patients with 25-OH vitamin D deficiency, are presented in Table 4.

**Table 4:** Comparison between patients with T2DM without vs. with 25-OH vitamin D deficiency.

Parameter	Without cholecalciferol deficiency (n = 58)	With cholecalciferol deficiency (n = 34)	p
Age (years) ^a^	58 [15]	61 [10]	0.092
Dialysis duration (years) ^a^	2 [2.8]	1 [3.1]	0.066
Weekly dialysis time (hours) ^a^	12 [4]	12 [0.5]	0.157
eKtV ^b^	1.31 ± 0.44	1.35 ± 0.50	0.701
BMI (kg/m^2^) ^b^	26.3 ± 4.7	26.4 ± 5.0	0.925
Hemoglobin (g/dL) ^b^	11.5 ± 1.4	11.0 ± 1.8	0.090
Ferritin (ng/mL) ^a^	478.5 [309]	625 [557]	0.214
TSAT (%)^a^	16 [33.8]	15.0 [32.0]	0.401
hsCRP (mg/dL)^a^	2.0 [5.0]	3.5 [13.0]	0.273
Albumin (g/dL) ^b^	3.85 ± 0.75	3.56 ± 0.70	0.073
Ca (mg/dL) ^b^	8.41 ± 0.87	8.54 ± 1.12	0.524
PO4 (mg/dL) ^b^	5.73 ± 1.76	5.33 ± 1.66	0.294
CaxPO4 ^b^	48.7 ± 15.2	45.3 ± 13.0	0.274
HCO3 ^b^	20.6 ± 3.9	19.4 ± 5.1	0.209
iPTH ^a^	261.4 [491.1]	227.5 [356.3]	0.502
ALP ^a^	101 [70]	91 [75]	0.900
PTX (%) ^*c*^	2 (3.4%)	2 (5.9%)	0.581
FAV (%) ^*c*^	46 (79.3%)	26 (76.5%)	0.750
Mortality (%)^*c*^	14 (24.1%)	17(50.0%)	0.011*
Calcitriol supplementation (%) ^c^	12 (20.7%)	12 (35.3%)	0.124

*Differences are significant

^a^ Distributions are not Gaussian. Data is presented as median and [interquartile range]; p was calculated with Mann-Whitney U test.

^b^ Data is presented as mean±S; p was calculated with t-student test.

^c^ Data is presented as number and (percentage of total). p was calculated using chi-square test.

Continuous variables distributions were tested for normality using Shapiro-Wilk test and for equality of variances with Levene’s test.

**Fig 2:**
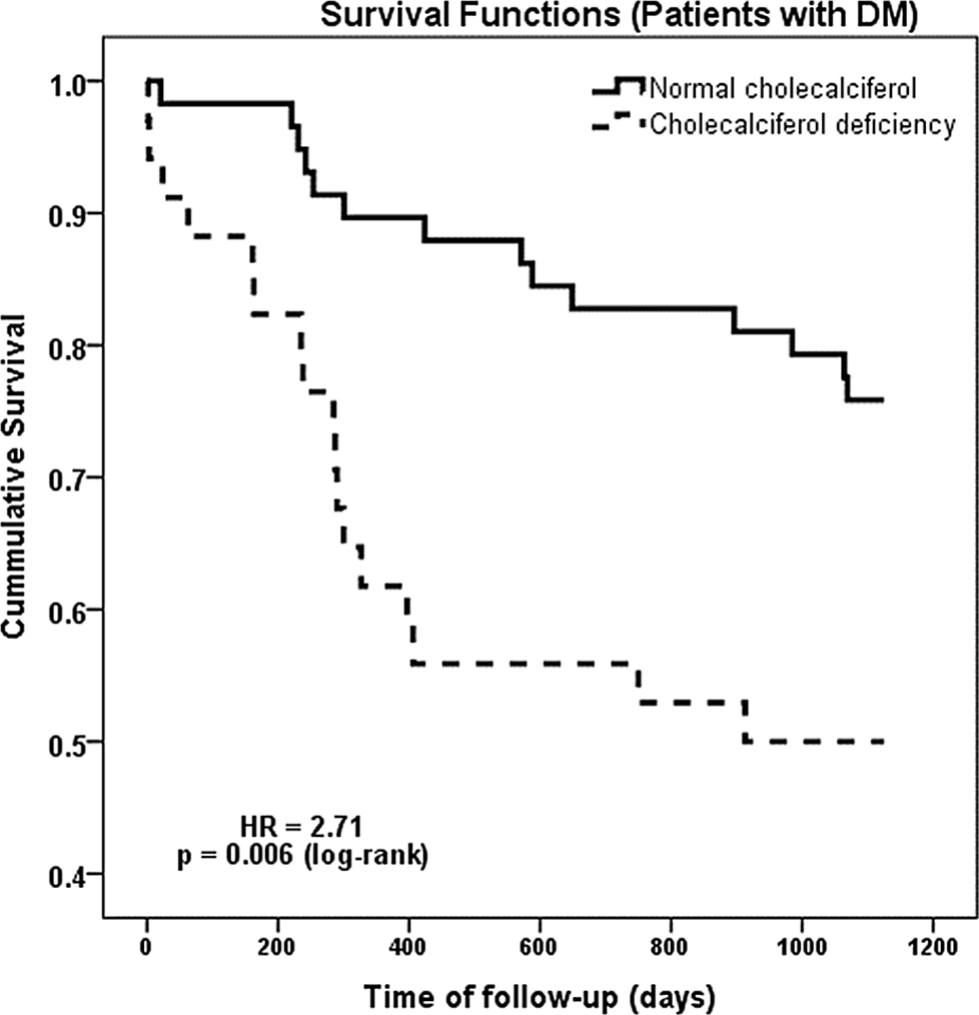
Survival analysis in dialyzed patients with DM.

The presence of DM in dialyzed patient was associated with an increase prevalence of 25-OH vitamin D deficiency (37.0% vs. 24.0%; p = 0.009). The patients with DM and 25-OH vitamin D deficiency had an increased probability to die in the three years of follow-up (50.0% vs. 24.1%; p = 0.011). The survival analysis revealed a worsening prognosis associated with vitamin D deficiency—HR = 2.71, [1.34 to 5.52]95%CI; p = 0.006, (Fig 2).

In order to assess the involvement of multiple factors in the 3 years risk of death for dialyzed patients with DM, a multiple, backward conditional (stepwise, acceptance threshold p<0.1, exclusion threshold p>0.2) Cox proportional hazards model was built, having the following co-factors: age, dialysis duration, BMI, Hemoglobin, serum albumin concentration, 25-OH vitamin D levels, the presence of associated co-morbidities (DM, stroke, CAD and PVD) and infection with any of the B and C hepatic viruses. The stepwise algorithm in the Cox model accepted the following predictors: age, BMI, 25-OH vitamin D deficiency (dichotomous), CAD (dichotomous) and PVD (dichotomous). The resulted model revealed significant influence on the risk of death in the 3 years of follow-up for 25-OH vitamin D deficiency (HR = 1.71, p = 0.003), age (HR = 1.02 per one year increase, p = 0.004), CAD (HR = 1.55, p = 0.046) and PVD (HR = 1.50, p = 0.029). The results of the accepted model are presented in Table 5.

**Table 5:** Predictors accepted in the Cox proportional-hazards model.

Predictor	B	Wald	Hazard Ratio [95% CI]	p
Age (per one year)	0.02	8.210	1.02 [1.01–1.03]	0.004 *
BMI (per 1 kg/m^2^)	-0.034	3.076	0.97 [0.93–1.01]	0.079
25-OH vitamin D deficiency	0.538	9.075	1.71 [1.21–2.43]	0.003 *
CAD	0.441	3.762	1.55 [1.02–2.41]	0.046 *
PVD	0.405	4.779	1.50 [1.04–2.16]	0.029 *

* predictor is significant

## Discussion

According to our knowledge this paper is the first East European study evidencing the fact that HD patients with DM have high prevalence of vitamin D deficiency associated with an important burden of mortality.

At enrolment the prevalence of DM was 15.3% (being less than the recently published prevalence for The East European countries in the COSMOS study—24.1%) [23]. (In the 3 years of follow-up, the prevalence increased to 22.2%). The 3 years mortality in our cohort was very low as compared to The ERA-EDTA data [24], probably related to the low median age of the investigated cohort, being higher in patients with DM. The mortality risk was also significantly higher in the DM group. Similar results have been published in the DOPPS study on Japanese patients [25] and in the NECOSAD and HEMO studies [26, 27]. Moreover, Schroijen found similar mortality risk in HD patients from NECOSAD having diabetic nephropathy as primary disease as well as in those having DM as co-morbid condition. The mortality risk seems to be also increased in the peritoneal dialysis DM patients as compared to no DM ones [28].

The high mortality excess in patients with CKD/ ESRD and DM could be attributed to the amplified and not summed-up effects of risk factors of both DM and CKD and, as a consequence, to the amplified and not summed-up end organ damage accumulation in both diseases [26,29].

In this study 25-OH vitamin D was used to estimate the vitamin D status of the patients and vitamin D deficiency was defined as 25 (OH) D levels < 12 ng/ml [19]. We found an increased prevalence of vitamin D deficiency in DM patients as compared to no DM ones. Higher prevalence of vitamin D deficiency in DM associated with CKD was evidenced both in pre dialysis CKD [15, 16, 30] as well as in patients undergoing renal replacement therapy [31, 32] and it was attributed to urinary loss or resistance to vitamin D replacement therapy [15, 33]. However the results published in the recent years are heterogeneous because of the use of different cut-off levels for the definition of vitamin D deficiency. Thus, French cohort of prevalent HD patients, Jean et al. use for 25-OH- vitamin D deficiency a cut-off level of 18 ng/ml) [32]. Anand et al. found a median serum 25-OH vitamin D concentration of 12.9 ng/ml in their dialysed cohort and considered severe vitamin D deficiency at 25-OH vitamin D serum levels below 10,6 ng/ml [34]. In diabetic HD patients, Drechsler et al used for severely vitamin D deficiency cut-off values of <10.04 ng/ml and for moderately vitamin D deficiency > 10.04 ng/ml and < = 20.08 ng/ml [31]. Del Valle et al considered vitamin D deficiency levels of 25-OH vitamin D below 15 ng/ml, in patients with CKD stage 5 on hemodialysis [35]. Bansal et al considered vitamin D deficiency in hemodialysis patients at cut-off levels of 25-OH vitamin D the values < 20 ng/ml respectively for severe vitamin D deficiency levels below 10 ng/ml [36]. Barreto et al, consider in CKD patients that vitamin D deficiency is characterized by 25-OH D levels < 15 ng/ml [37]. In the Accelerated Mortality on Renal Replacement (ArMORR) cohort study of incident chronic hemodialysis patients, the authors used a threshold for vitamin D deficiency of 30 ng/ml [38].

In this study the three year mortality was significantly higher in the DM group. The survival analysis revealed a worsening prognosis associated with vitamin D deficiency (HR = 2.71 p = 0.006). In the 3 years follow-up of our cohort in DM patients the mortality risk was significantly influenced by 25-OH vitamin D deficiency, age, CAD and PVD.

There are few data about vitamin D deficiency and the risk of death in ESRD patients with DM on HD therapy [31]. However there is much information about low vitamin D levels and increased all cause and or cardiovascular mortality risk in the general population [39, 40], pre dialysis CKD [41, 42] and ESRD on HD therapy [13, 34]. Moreover vitamin D therapy in deficient general population, in pre dialysis CKD patients and in ESRD patients on HD therapy reduced cardiovascular and all-cause mortality [43, 44]. The vitamin D effects on mortality have been attributed to the interference on cell proliferation and differentiation, immune cell function, and some of tissue-specific processes [45]. Due to these interventions, experimental and clinical studies tend to demonstrate cardioprotective effects by regulating atherogenesis (inhibiting macrophage to foam cell transformation) and by reducing left ventricular hypertrophy. The neuroprotective effect of vitamin D was attributed to diminished thrombogenesis and up regulation of insulin-like growth factor 1 synthesis [46]. Also, there are data about renoprotective actions of vitamin D by reducing fibrosis, apoptosis and inflammation through suppressing the NF-kB pathway and expression of pro inflammatory and pro fibrotic cytokines [47, 48].Moreover, vitamin D suppresses renin-angiotensin-aldosterone system activity by inhibiting renin synthesis and by reducing the expression AT1 receptors in the kidney [49, 50]. These actions could lead to cardiac and nephroprotective effects by modulating blood pressure, reducing proteinuria and decreasing progression of CKD [47]. In patients with DM vitamin D attenuates the effects of advanced glycation end products (AGEs) on vessel walls, thus reducing endothelial dysfunction and arterial stiffness [51].

In our cohort the 3 years mortality was not influenced by Calcium, Phosphate, ALF, and PTH levels supporting the idea that vitamin D deficiency may influence mortality independent of iPTH as it was pointed out by Bouillon et al. and more recently by Folsom et al. [52, 53]. In our study the PTH levels were significantly lower in DM patients. Lower levels of PTH, in both predialysis CKD as well as in HD treated DM patients, have been related to poor glycemic control (hyperglycemia having an inhibitory effect of on PTH cells) or high levels of AGEs inducing suppression of PTH synthesis [54, 55, 56].

The observational design of our study does not allow conclusions concerning a direct causal relation between vitamin D deficiency and the excess mortality in DM patients on HD therapy. One should take in account also the fact that in a recent study it was suggested that VDR polymorphism interacting with 25-OH vitamin D levels may determine disease susceptibility and major clinical outcome in all vitamin D deficient patients [57].

On the other hand vitamin D deficiency could be considered a marker of illness severity, being a result of low provitamin D synthesis in the skin due to reduced solar exposure. Severely ill patients tend to be less active and tend to avoid solar exposure and in advanced CKD, in particular, malnutrition may also contribute to this status [58].

Our study has some strengths and limitations. It investigates a significant cohort of East European HD patients for a long period of follow-up time (3 years). The statistical analysis permitted us to demonstrate that vitamin D deficiency is an independent predictor of mortality in HD patients with DM. The main limitations could be considered the lack of information about some baseline data (physical activity, sun exposure, diet), the lack of data about fibroblast growth factor 23 levels, and the vitamin D receptor polymorphism.

## Conclusions

In summary, our prospective follow-up study shows that severe vitamin D deficiency (baseline levels of 25-OH vitamin D < 12 ng/ml) predict increased risk of all-cause mortality in HD patients with DM. Vitamin D levels correction may reduce the high added mortality risk in these patients.

## References

[1] Collins AJ, Foley RN, Chavers B, Gilbertson D, Herzog C, Ishani A, et al US Renal Data System 2013 Annual Data Report. Am J Kidney Dis. 2014;63(1 Suppl):A7 10.1053/j.ajkd.2013.11.001 24360288

[2] Hill CJ, Fogarty DG. Changing trends in end-stage renal disease due to diabetes in the United Kingdom. J Ren Care. 2012;38 Suppl 1:12–22 10.1111/j.1755-6686.2012.00273.x 22348360

[3] Levin A, Li YC. Vitamin D and its analogues: do they protect against cardiovascular disease in patients with kidney disease? Kidney Int. 2005;68(5):1973–81. 16221197 10.1111/j.1523-1755.2005.00651.x

[4] Cheung AK, Sarnak MJ, Yan G, Dwyer JT, Heyka RJ, Rocco MV, et al Atherosclerotic cardiovascular disease risks in chronic hemodialysis patients. Kidney Int. 2000; 58(1):353–62. 10886582 10.1046/j.1523-1755.2000.00173.x

[5] Sarnak MJ, Coronado BE, Greene T, Wang SR, Kusek JW, Beck GJ, et al Cardiovascular disease risk factors in chronic renal insufficiency. Clin Nephrol. 2002;57(5):327–35. 12036190 10.5414/cnp57327

[6] Ravani P, Malberti F, Tripepi G, Pecchini P, Cutrupi S, Pizzini P, et al Vitamin D levels and patient outcome in chronic kidney disease. Kidney Int. 2009;75(1):88–95 10.1038/ki.2008.501 18843258

[7] Wang L, Song Y, Manson JE, Pilz S, März W, Michaëlsson K, et al Circulating 25-hydroxy-vitamin D and risk of cardiovascular disease: a meta-analysis of prospective studies. Circ Cardiovasc Qual Outcomes. 2012;5:819–29. 10.1161/CIRCOUTCOMES.112.967604 23149428 PMC3510675

[8] Zittermann A, Iodice S, Pilz S, Grant WB, Bagnardi V, Gandini S. Vitamin D deficiency and mortality risk in the general population: a meta-analysis of prospective cohort studies. Am J Clin Nutr. 2012;95:91–100. 10.3945/ajcn.111.014779 22170374

[9] Fraser A, Williams D, Lawlor DA. Associations of serum 25-hydroxyvitamin D, parathyroid hormone and calcium with cardiovascular risk factors: analysis of 3 NHANES cycles (2001–2006). PLoS One. 2010;5(11):e13882 10.1371/journal.pone.0013882 21085485 PMC2976699

[10] Krassilnikova M, Ostrow K, Bader A, Heeger P, Mehrotra A. Low Dietary Intake of Vitamin D and Vitamin D Deficiency in Hemodialysis Patients. J Nephrol Ther. 2014;4(3). 25068077 10.4172/2161-0959.1000166 PMC4109326

[11] London GM, Guérin AP, Verbeke FH, Pannier B, Boutouyrie P, Marchais SJ, et al Mineral metabolism and arterial functions in end-stage renal disease: potential role of 25-hydroxyvitamin D deficiency. J Am Soc Nephrol. 2007;18:613–20. 17202417 10.1681/ASN.2006060573

[12] Matias PJ, Ferreira C, Jorge C, Borges M, Aires I, Amaral T, et al 25-Hydroxyvitamin D3, arterial calcifications and cardiovascular risk markers in haemodialysis patients. Nephrol Dial Transplant. 2009; 24:611–8 10.1093/ndt/gfn502 18775809

[13] Drechsler C, Pilz S, Obermayer-Pietsch B, Verduijn M, Tomaschitz A, Krane V, et al Vitamin D deficiency is associated with sudden cardiac death, combined cardiovascular events, and mortality in haemodialysis patients. Eur Heart J. 2010;31:2253–61. 10.1093/eurheartj/ehq246 20688781 PMC2938469

[14] Bucharles S, Barberato SH, Stinghen AE, Gruber B, Meister H, Mehl A, et al Hypovitaminosis D is associated with systemic inflammation and concentric myocardial geometric pattern in hemodialysis patients with low iPTH levels. Nephron Clin Pract. 2011;118:c384–91. 10.1159/000323664 21325871

[15] Mehrotra R, Kermah D, Budoff M, Salusky IB, Mao SS, Gao YL, et al Hypovitaminosis D in chronic kidney disease. Clin J Am Soc Nephrol. 2008;3:1144–51. 10.2215/CJN.05781207 18417740 PMC2440286

[16] Ureña-Torres P, Metzger M, Haymann JP, Karras A, Boffa JJ, Flamant M, et al Association of kidney function, vitamin D deficiency, and circulating markers of mineral and bone disorders in CKD. Am J Kidney Dis. 2011; 58:544–53 10.1053/j.ajkd.2011.04.029 21803465

[17] Joergensen C, Gall MA, Schmedes A, Tarnow L, Parving HH, Rossing P. Vitamin D Levels and Mortality in Type 2 Diabetes Diabetes Care. 2010; 33: 2238–2243. 10.2337/dc10-0582 20606205 PMC2945166

[18] Joergensen C, Hovind P, Schmedes A, Parving HH, Rossing P. Vitamin D levels, microvascular complications, and mortality in type 1 diabetes. Diabetes Care. 2011;34:1081–5 10.2337/dc10-2459 21525501 PMC3114500

[19] IOM (Institute of Medicine). 2011 Dietary Reference Intakes for Calcium and Vitamin D. Washington, DC: The National Academies Press 21796828

[20] Schöttker B, Haug U, Schomburg L, Köhrle J, Perna L, Müller H, et al Strong associations of 25-hydroxyvitamin D concentrations with all-cause, cardiovascular, cancer, and respiratory disease mortality in a large cohort study. Am J Clin Nutr. 2013;97(4):782–93. 10.3945/ajcn.112.047712 23446902

[21] Holick MF, Binkley NC, Bischoff-Ferrari HA, Gordon CM, Hanley DA, Heaney RP, et al Evaluation, treatment, and prevention of vitamin D deficiency: an Endocrine Society clinical practice guideline. J Clin Endocrinol Metab. 2011;96(7):1911–30. 10.1210/jc.2011-0385 21646368

[22] Brun E, Nelson RG, Bennett PH, Imperatore G, Zoppini G, Verlato G, et al Diabetes duration and cause-specific mortality in the Verona Diabetes Study. Diabetes Care. 2000;23(8):1119–23. 10937508 10.2337/diacare.23.8.1119

[23] Fernández-Martín JL, Carrero JJ, Benedik M, Bos WJ, Covic A, Ferreira A, et al COSMOS: the dialysis scenario of CKD-MBD in Europe. Nephrol Dial Transplant. 2013;28:1922–35 10.1093/ndt/gfs418 23166310

[24] ERA-EDTA Registry: ERA-EDTA Registry Annual Report 2012. Academic Medical Center, Department of Medical Informatics, Amsterdam, The Netherlands, 2014; ISBN 817480-4-9

[25] Hayashino Y, Fukuhara S, Akiba T, Akizawa T, Asano Y, Saito A, et al Diabetes, glycaemic control and mortality risk in patients on haemodialysis: the Japan Dialysis Outcomes and Practice Pattern Study. Diabetologia. 2007;50:1170–7. 17393134 10.1007/s00125-007-0650-z

[26] Sattar A, Argyropoulos C, Weissfeld L, Younas N, Fried L, Kellum JA, et al All-cause and cause-specific mortality associated with diabetes in prevalent hemodialysis patients. BMC Nephrol. 2012;1;13:130 10.1186/1471-2369-13-130 23025844 PMC3519533

[27] Schroijen MA, Dekkers OM, Grootendorst DC, Noordzij M, Romijn JA, Kredier RT, et al Survival in dialysis patients is not different between patients with diabetes as primary renal disease and patients with diabetes as a co-morbid condition. BMC Nephrol. 2011; 9;12:69 10.1186/1471-2369-12-69 22182634 PMC3259092

[28] Ozener C, Arikan H, Karayaylali I, Utas C, Bozfakioglu S, Akpolat T, et al The impact of diabetes mellitus on peritoneal dialysis: the Turkey Multicenter Clinic Study. Ren Fail. 2014;36:149–53 10.3109/0886022X.2013.843275 24131086

[29] Afkarian M, Sachs MC, Kestenbaum B, Hirsch IB, Tuttle KR, Himmelfarb J, et al Kidney disease and increased mortality risk in type 2 diabetes. J Am Soc Nephrol. 2013;24:302–8. 10.1681/ASN.2012070718 23362314 PMC3559486

[30] Echida Y, Mochizuki T, Uchida K, Tsuchiya K, Nitta K. Risk factors for vitamin D deficiency in patients with chronic kidney disease. Intern Med. 2012;51:845–50 22504237 10.2169/internalmedicine.51.6897

[31] Drechsler C, Verduijn M, Pilz S, Dekker FW, Krediet RT, Ritz E, et al Vitamin D status and clinical outcomes in incident dialysis patients: results from the NECOSAD study. Nephrol Dial Transplant. 2011;26(3):1024–32. 10.1093/ndt/gfq606 20947538

[32] Jean G, Lataillade D, Genet L, Legrand E, Kuentz F, Moreau-Gaudry X, et al Impact of hypovitaminosis D and alfacalcidol therapy on survival of hemodialysis patients: results from the French ARNOS study. Nephron Clin Pract. 2011;118:c204–10 10.1159/000321507 21178377

[33] Alshayeb HM, Wall BM, Showkat A, Mangold T, Quarles LD. Chronic kidney disease and diabetes mellitus predict resistance to vitamin D replacement therapy. Am J Med Sci. 2013; PubMed 23221508 10.1097/MAJ.0b013e31826af2d3 PMC6350532

[34] Anand S, Kaysen GA, Chertow GM, Johansen KL, Grimes B, Dalrymple LS, et al Vitamin D deficiency, self-reported physical activity and health-related quality of life: the Comprehensive Dialysis Study. Nephrol Dial Transplant. 2011;26:3683–8. 10.1093/ndt/gfr098 21430182 PMC3247798

[35] Del Valle E, Negri AL, Aguirre C, Fradinger E, Zanchetta JR. Prevalence of 25(OH) vitamin D insufficiency and deficiency in chronic kidney disease stage 5 patients on hemodialysis. Hemodial Int. 2007;11(3):315–21. 17576296 10.1111/j.1542-4758.2007.00186.x

[36] Bansal B, Bansal S, Mithal A, Kher V, Marwaha R. Vitamin D deficiency in hemodialysis patients. Indian J Endocrinol Metab. 2012;16(2):270–3. 10.4103/2230-8210.93749 22470866 PMC3313747

[37] Barreto DV, Barreto FC, Liabeuf S, Temmar M, Boitte F, Choukroun G, et al Vitamin D affects survival independently of vascular calcification in chronic kidney disease. Clin J Am Soc Nephrol. 2009;4(6):1128–35. 10.2215/CJN.00260109 19443628 PMC2689889

[38] Bhan I, Burnett-Bowie SA, Ye J, Tonelli M, Thadhani R. Clinical measures identify vitamin D deficiency in dialysis. Clin J Am Soc Nephrol. 2010;5:460–7 10.2215/CJN.06440909 20185603 PMC2827576

[39] Schöttker B, Jorde R, Peasey A, Thorand B, Jansen EH, Groot Ld et al Vitamin D and mortality: meta-analysis of individual participant data from a large consortium of cohort studies from Europe and the United States. BMJ. 2014; 348: g3656 10.1136/bmj.g3656 24938302 PMC4061380

[40] Sempos CT, Durazo-Arvizu RA, Dawson-Hughes B, Yetley EA, Looker AC, Schleicher RL, et al Is there a reverse J-shaped association between 25-hydroxyvitamin D and all-cause mortality? Results from the U.S. nationally representative NHANES. J Clin Endocrinol Metab. 2013;98:3001–9 10.1210/jc.2013-1333 23666975 PMC3701270

[41] Mehrotra R, Kermah DA, Salusky IB, Wolf MS, Thadhani RI, Chiu YW, et al Chronic kidney disease, hypovitaminosis D, and mortality in the United States. Kidney Int. 2009;76:977–83 10.1038/ki.2009.288 19657329 PMC3791220

[42] Pilz S, Iodice S, Zittermann A, Grant WB, Gandini S. Vitamin D status and mortality risk in CKD: a meta-analysis of prospective studies. Am J Kidney Dis. 2011;58:374–82. 10.1053/j.ajkd.2011.03.020 21636193

[43] Chowdhury R, Kunutsor S, Vitezova A, Oliver-Williams C, Chowdhury S, Kiefte-de-Jong JC, et al Vitamin D and risk of cause specific death: systematic review and meta-analysis of observational cohort and randomized intervention studies. BMJ. 2014;1;348:g1903 10.1136/bmj.g1903 24690623 PMC3972416

[44] Zheng Z, Shi H, Jia J, Li D, Lin S. Vitamin D supplementation and mortality risk in chronic kidney disease: a meta-analysis of 20 observational studies. BMC Nephrol. 2013;14:199 10.1186/1471-2369-14-199 24066946 PMC3851063

[45] de Boer IH, Thadhani R. Vitamin D deficiency: consequence or cause of CKD? Clin J Am Soc Nephrol. 2013;8:1844–6. 10.2215/CJN.09480913 24135217 PMC3817913

[46] Balden R, Selvamani A, Sohrabji F. Vitamin D deficiency exacerbates experimental stroke injury and dysregulates ischemia-induced inflammation in adult rats. Endocrinology. 2012; 153:2420–35. 10.1210/en.2011-1783 22408173 PMC3339639

[47] Lang CL, Wang MH, Chiang CK, Lu KC. Vitamin D and the Immune System from the Nephrologist's Viewpoint. ISRN Endocrinol. 2014; 2014:1054–56. 10.1155/2014/105456 PMC3920624 24587915

[48] Achinger SG, Ayus JC. The role of vitamin D in left ventricular hypertrophy and cardiac function. Kidney Int Suppl. 2005;(95):S37–42. 15882312 10.1111/j.1523-1755.2005.09506.x

[49] Li YC. Vitamin D in chronic kidney disease. Contrib Nephrol. 2013;180:98–109 10.1159/000346789 23652553

[50] Canale D, de Bragança AC, Gonçalves JG, Shimizu MH, Sanches TR, Andrade L et al Vitamin D Deficiency Aggravates Nephrotoxicity, Hypertension and Dyslipidemia Caused by Tenofovir: Role of Oxidative Stress and Renin-Angiotensin System PLoS One. 2014; 9(7): e103055 10.1371/journal.pone.0103055 25048368 PMC4105615

[51] Al Mheid I, Patel R, Murrow J, Morris A, Rahman A, Fike L, et al Vitamin D status is associated with arterial stiffness and vascular dysfunction in healthy humans. J Am Coll Cardiol. 2011;58:186–92. 10.1016/j.jacc.2011.02.051 21718915 PMC3896949

[52] Bouillon R, Carmeliet G, Verlinden L, van Etten E, Verstuyf A, Luderer HF, et al Vitamin D and human health: lessons from vitamin D receptor null mice. Endocr Rev. 2008;29:726–76 10.1210/er.2008-0004 18694980 PMC2583388

[53] Folsom AR, Alonso A, Misialek JR, Michos ED, Selvin E, Coresh J, et al Parathyroid hormone concentration and risk of cardiovascular diseases: The Atherosclerosis Risk in Communities (ARIC) study. Am Heart J. 2014;168:296–302. 10.1016/j.ahj.2014.04.017 25173540 PMC4150218

[54] Wahl P, Xie H, Scialla J, Anderson CA, Bellovich K, Brecklin C, et al Earlier onset and greater severity of disordered mineral metabolism in diabetic patients with chronic kidney disease. Diabetes Care. 2012;35:994–1001. 10.2337/dc11-2235 22446176 PMC3329844

[55] Dan S, Aditya P, Samanta M, Jothimalar R, Soundarajan P. Effect of glycemic control on intact parathyroid hormone level in end stage renal disease patients on maintenance hemodialysis. Diabetes Res Clin Pract. 2014. pii:S0168-8227(14)00180-6. 10.1016/j.diabres.2014.04.002 25015316

[56] Murakami R, Murakami S, Tsushima R, Ueda C, Mikami K, Ebina T, et al Glycaemic control and serum intact parathyroid hormone levels in diabetic patients on haemodialysis therapy. Nephrol Dial Transplant.2008;23:315–20 17956892 10.1093/ndt/gfm639

[57] Levin GP, Robinson-Cohen C, de Boer IH, Houston DK, Lohman K, Liu Y et al Genetic variants and associations of 25-hydroxyvitamin D concentrations with major clinical outcomes. JAMA. 2012 11 14;308:1898–905. 10.1001/jama.2012.17304 23150009 PMC3645444

[58] Autier P, Boniol M, Pizot C, Mullie P. Vitamin D status and ill health: a systematic review. Lancet Diabetes Endocrinol. 2014;2:76–89 10.1016/S2213-8587(13)70165-7 24622671

